# Test-Retest Reliability of a New Medial Temporal Atrophy Morphological Metric

**DOI:** 10.1155/2012/979804

**Published:** 2012-09-17

**Authors:** Simon Duchesne, Fernando Valdivia, Abderazzak Mouiha, Nicolas Robitaille

**Affiliations:** ^1^Département de Radiologie, Faculté de Médecine, Université Laval, Quebec, QC, Canada G1V 0A6; ^2^Institut Universitaire en Santé Mentale de Québec, Quebec, QC, Canada G1J 2G3

## Abstract

Clinicians and researchers alike are in need of quantitative and robust measurement tools to assess medial temporal lobe atrophy (MTA) due to Alzheimer's disease (AD). We recently proposed a morphological metric, extracted from T1-weighted magnetic resonance images (MRI), to track and estimate MTA in cohorts of controls, AD, and mild cognitive impairment subjects, at high-risk of progression to dementia. In this paper, we investigated its reliability through analysis of within-session scan/repeat images and scan/rescans from large multicenter studies. In total, we used MRI data from 1051 subjects recruited at over 60 centers. We processed the data identically and calculated our metric for each individual, based on the concept of distance in a high-dimensional space of intensity and shape characteristics. Over 759 subjects, the scan/repeat change in the mean was 1.97% (SD: 21.2%). Over three subjects, the scan/rescan change in the mean was 0.89% (SD: 22.1%). At this level, the minimum trial size required to detect this difference is 68 individuals for both samples. Our scan/repeat and scan/rescan results demonstrate that our MTA assessment metric shows high reliability, a necessary component of validity.

## 1. Introduction

Early detection of Alzheimer's dementia (AD), critical for treatment success, is a high-priority research area. The development of disease-modifying treatment strategies requires objective characterization techniques and quantitative biomarkers able to identify AD with higher accuracy and at a much earlier stage than clinically based assessment [1]. Given that structural magnetic resonance imaging (MRI) (e.g., T1 weighted) on 1 to 3 Tesla clinical scanners allows the *in vivo *assessment of changes such as medial temporal lobe atrophy (MTA) due to AD, it has been proposed to fulfill the role of quantitative biomarkers in recent reports [2, 3]. 

We have developed a sophisticated automated image processing method for the purpose of evaluating MTA in the context of AD. We recently proposed a single, high-dimensional morphological metric called the disease evaluation factor (DEF) extracted from T1-weighted MRI and able to track and estimate disease state [4]. In our previous report we provided estimates of this metric's efficiency at the discrimination of cognitively normal, control subjects (CTRL) from probable AD patients, as well as the prediction of conversion in mild cognitive impairment (MCI) subjects to probable AD. 

Thorough technique verification, validation, and evaluation are necessary, however, in order for imaging biomarkers such as the DEF to be used in clinical trials enrichment, and more importantly, as a diagnostic aid to community physicians. As an essential component of the verification process, comprehensive metrological investigation of MRI-based metrics must include reliability testing.

Reliability is an important component of the precision of a measurement and relates to the consistency of measurements taken by a single person or instrument on the same item and under the same conditions. A less-than-perfect test-retest reliability causes test-retest variability, reducing confidence in the result and decreasing the test's statistical power. Reliability testing is particularly important for MRI-based metrics, which, while acquired with similar protocols, will show dissimilar intensity contrasts for the same tissue types [5]. These systematic and random variations are machine dependent and can be corrected for the most part via image denoising [6], bias field inhomogeneity estimation [7], and intensity standardization [8].

In this paper we investigated the reliability of our DEF metric through analysis of cross-sectional (i.e., one timepoint) scan/repeat scan and scan/rescan images from two multicentric studies. First, we took advantage of the fact that subjects in the Alzheimer's Disease Neuroimaging Initiative (ADNI) study received two within-session T1-weighted scans at their baseline visit to test for scan/repeat scan analysis. Further, we employed data on three participants in the Pilot European ADNI that had been scanned at seven different sites in a short timeframe to test for Scan/Rescan reliability. We report minimum clinical trial sample size increases at various different levels based on the calculated detection threshold.

Reliability analysis is an important, necessary, and often overlooked step between bench and bedside in the research and clinical contexts. 

## 2. Materials and Methods

### 2.1. Ethics

Institutional review boards of all participating institutions approved the procedures for this study. Written informed consent was obtained from all participants or surrogates. More information about ADNI ^1^ and Pilot European ADNI investigators are provided in the Acknowledgments.

### 2.2. Subjects

In this study we used data from three different studies, totaling 1051 subjects from over 60 centers.

- The first was the *Mapping group*, consisting of 145 young control subjects from the International Consortium for Brain Mapping database [9].
- The second was the *Classification group*, which consisted in 70 probable AD and 69 CTRL subjects from the LENITEM database [10]. We required those first two groups to build our high-dimensional metric;
- The third was the *Scan/Repeat Test Group*, which consisted in 1518 baseline MRIs (scan + same-session repeat scans) from 759 CTRL, MCI, and probable AD subjects participating in ADNI, acquired on more than 50 different 1.5T scanners using a similar 3D T1-weighted MP-RAGE protocol [11]. Inclusion criteria to the ADNI study were as follows.
  - CTRL are MMSE scores [12] between 24–30 (inclusive), a CDR [13] of 0, nondepressed, non-MCI, and nondemented. The age range of normal subjects was roughly matched to that of MCI and mild AD subjects.
  - MCI subjects are MMSE scores between 24–30 (inclusive), a memory complaint, objective memory loss measured by education adjusted scores on Wechsler Memory Scale Logical Memory II [14], a CDR of 0.5, absence of significant levels of impairment in other cognitive domains, essentially preserved activities of daily living, and an absence of dementia.
  - Mild AD is MMSE scores between 20–26 (inclusive), CDR of 0.5 or 1.0, and meets NINCDS/ADRDA criteria for probable AD [15].
-  From the complete ADNI dataset of 822 subjects at baseline, we selected individuals for the *Scan/Repeat Test Group* that had both valid entry images and processed images that passed *automated *quality control [16].
- Finally, the fourth was the *Scan/Rescan Test Group*, which was obtained with permission from the multi-centric Pilot European ADNI project [17]. It included data from three healthy volunteers acting as human quality control phantoms for the study.

### 2.3. MRI Acquisitions

Subjects in the *Mapping group *were scanned in Montreal, QC, Canada on a Philips Healthcare Gyroscan 1.5T scanner (Best, The Netherlands) using a T1-weighted fast gradient echo sequence (sagittal acquisition, TR = 18 ms, TE = 10 ms, 1 × 1 × 1 mm^3^ voxels, flip angle 30°).

Subjects in the *Classification group *were scanned in Brescia, Italy on a single Philips Healthcare Gyroscan 1.0T scanner (Best, The Netherlands) using a T1-weighted fast field echo sequence (sagittal acquisition, TR = 25 ms, TE = 6.9 ms, 1 × 1 × 1, 3 mm^3^ voxels). 

Subjects in the *Scan/Repeat Test Group *were scanned on over 50 different 1.5T scanners (GE Medical Systems; Siemens Healthcare; Philips Healthcare) using a 3D T1-weighted MP-RAGE protocol or its equivalent [11]. In this protocol, within the same scan session, there were two 3D T1-weighted images acquired, allowing us to test reliability on this scan/repeat pair. The subject was not taken out of the scanner between acquisitions.

Subjects in the *Scan/Rescan Test Group* were scanned within the span of few weeks at seven different European centers (Sites 1 to 7), using the ADNI study 3D T1-weighted MP-RAGE protocol [11]. Six centers collected scan/rescan sessions, where the subject was taken out of the scanner between acquisitions. This allows us to estimate scan/rescan reliability on 18 comparison pairs.

### 2.4. Initial Image Processing

We processed all MRI volumes identically using the MINC image processing toolbox (http://www.bic.mni.mcgill.ca/ServicesSoftware/HomePage) and local software as follows: (a) noise removal [6]; (b) raw scanner intensity inhomogeneity correction [7]; (c) global registration (12 degrees of freedom) [18] to the reference image space defined by the BrainWeb T1-weighted image [19] (1-mm resolution, 0% noise, 0% nonuniformity), maximizing the mutual information between the two volumes [20]; (d) resampling to a 1-mm^3^ isotropic grid; (e) linear clamping to (0–100) intensity range; (f) intensity standardization [8]; (g) nonlinear registration of individual standardized subject images to the BrainWeb reference; (h) computation of determinants of the Jacobian of the deformation field [21].

### 2.5. High-Dimensional Metric

We generated a low-dimensional feature space with the *Mapping group *using Principal Components Analysis of (a) T1w MRI intensity z-score maps, as a proxy of tissue composition and (2) determinant maps, as a proxy of tissue atrophy. After computing components, data from the *Mapping group* were no longer used in the study.

We then projected intensity and determinant data from the *Classification group *into the space defined by the principal components and used a system of supervised linear classifiers with forward stepwise regression (*p-to-enter *0.05) to identify a restricted set of eigenvectors {*λ*_*f*_} forming a hyperplane that best separated the two classes under study (CTRL versus probable AD). After computing the classification function, data from the *Classification group* were no longer used in the study.

Finally, we projected *Test Group *data in the {*λ*_*f*_} eigenvector space. The morphological DEF metric is based on the concept of distance within the space defined by eigenvectors {*λ*_*f*_} [4]. Specifically, in this embodiment it consists in the calculated Mahalanobis distance (1) for each subject's image between the position *p* of a subject's image in the *Mapping group *feature space, along the restricted set of principal components, and the centroids of coordinates formed by the CTRL subjects of the *Classification group.*

The Mahalanobis distance between *p* and a group *G* is given by

(1) $$\begin{array}{c} \text{mahal}\left(p,G\right)=\sqrt{\left(p-\mu_{G}\right)S_{G}^{-1}\left(p-\mu_{G}\right)}, \end{array}$$

where *μ*_*G*_ and *S*_*G*_ are respectively the mean and covariance matrix of group *G*.

### 2.6. Experimental Design

We first tested reliability in the ADNI *Scan/Repeat Test Group*, that is, between within-session scan/repeat scan pairs, at a single study timepoint (namely, baseline scans). Secondly, we tested reliability in the Pilot European ADNI *Scan/Rescan Test Group*, that is, within-scanner scan/rescan pairs. For each reliability estimate, we calculated the change in the mean, standard deviation, and Pearson retest correlation. Finally, we estimated the impact of the reliability thresholds on the minimum trial size required to discriminate probable AD versus CTRL subjects, using conservative power assumptions, for cross-sectional evaluations.

## 3. Results

### 3.1. Scan/Repeat Scan Reliability

**Figure 1:**
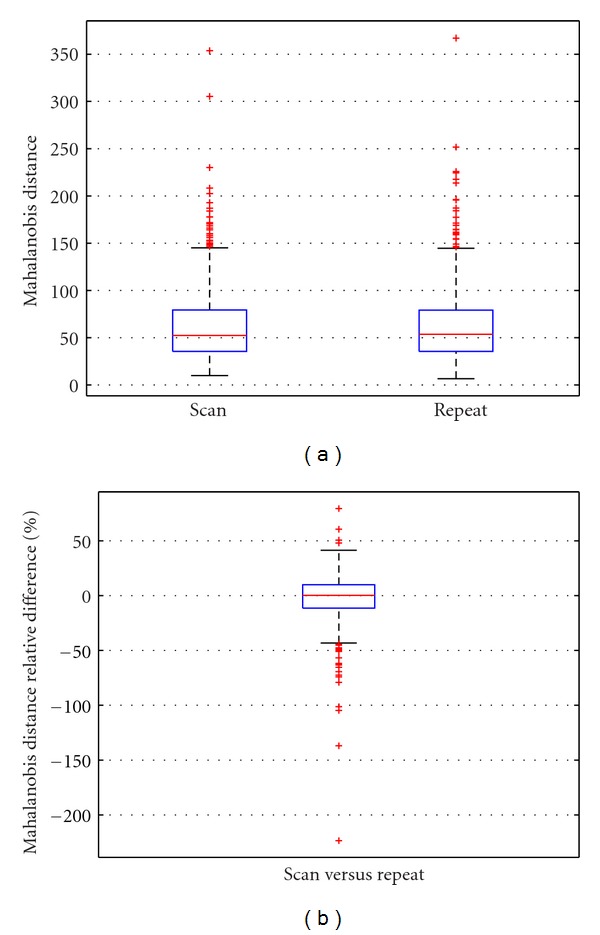
Absolute distances (a) and relative difference in % (b) for the DEF factor between within-session scan and repeat T1-weighted MR scans for 759 baseline ADNI subjects. The change in mean was 1.97%, with 95% confidence intervals 0.46%–3.48%.

Over the 759 subjects of the ADNI dataset, the scan/repeat change in the mean was 1.97% (95% CI: 0.46%–3.48%), with standard deviation 21.2% (*cf*.  Figure 1), and Pearson retest correlation *r* = 0.9381.

**Figure 2:**
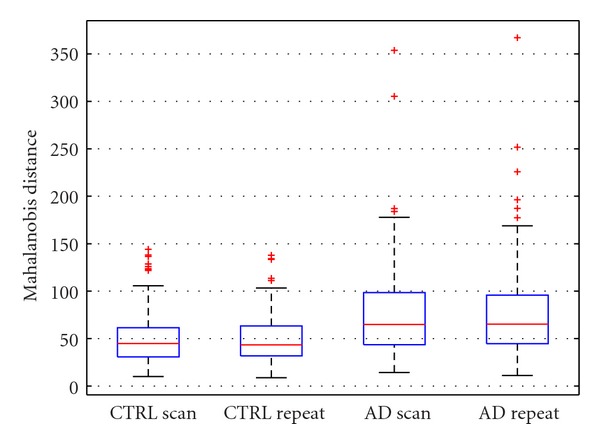
Absolute Mahalanobis distances (DEF factor) between scan/repeat scans for ADNI 203 CTRL subjects (left) and 169 probable AD subjects (right). While the between-group difference was significant, there were no statistical differences in reliability within each diagnostic group.

We ensured there were no statistical differences in reliability between scan/repeat scans in either CTRL or probable AD groups using the diagnostic provided by ADNI (*cf*.  Figure 2).

**Figure 3:**
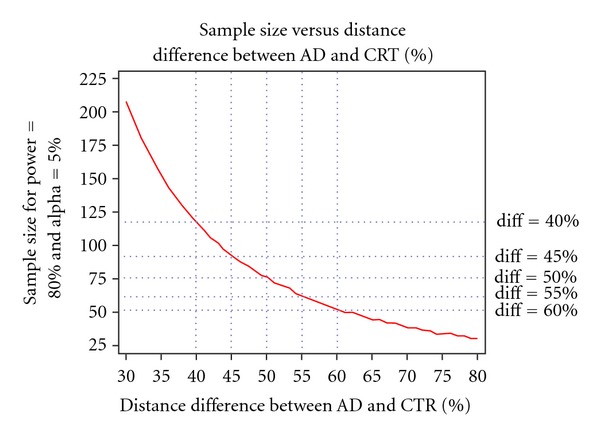
Sample sizes necessary to detect a given DEF difference (in %) between groups at 80% power and alpha level of 0.05.

As reported previously [4], the difference in DEF averages between probable AD and CTRL was 55%. At this level, the minimum trial size required to detect this difference is 62 individuals for both samples (*α* = 0.05; *β* = 0.50) (*cf*.  Figure 3). Due to the 1.97% minimum precision threshold of the technique, to reach identical power the trial size must increase to 68 individuals. 

**Figure 4:**
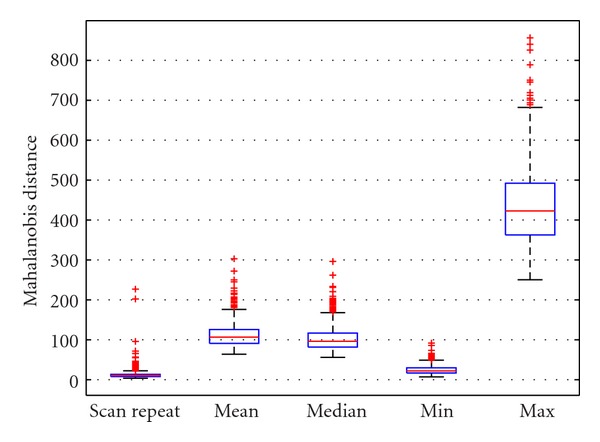
Comparison of scan/repeat scan distance versus all pairwise distances in the *Scan/Repeat Test Group *of 759 ADNI subjects shows that the closest image in the high-dimensional feature space remains its own repeat.

To evaluate whether the scan/repeat scan distance was smaller than the distance to any one image's nearest neighbor (scan or repeat), we proceeded by calculating all pairwise distances between subjects in the scan/repeat dataset. The comparison shows that the nearest neighbor in nearly all cases was the scan/repeat pair, as opposed to one of the possible neighbor (*cf*.  Figure 4). 

### 3.2. Scan/Rescan Reliability

**Figure 5:**
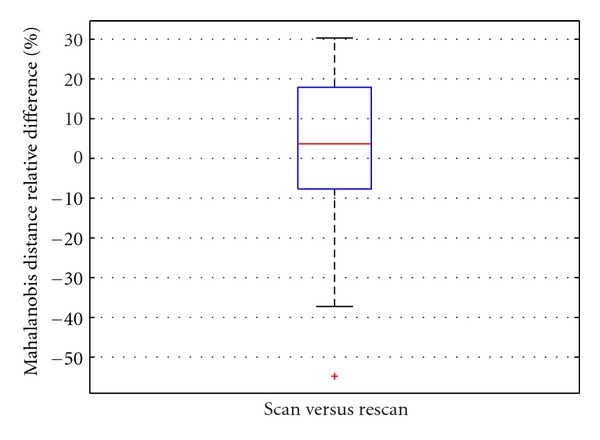
Relative difference in % for the DEF factor between different scan/rescan image pairs for Pilot European ADNI subjects (3 subjects at 6 sites; 18 scan/rescan pairs). The change in mean was 0.89%, with 95% confidence intervals (−14.34%, +12.56%).

Over the three subjects of the Pilot European ADNI dataset, the scan/rescan change in the mean was 0.89% (95% CI: −14.34%, +12.56%) (*cf*.  Figure 5), standard deviation 22.1%, and retest Pearson correlation *r* = 0.8609. Based on similar assumptions, the 0.89% precision threshold of the technique implies an increase in trial size from 62 to 64 individuals.

## 4. Discussion

Imaging biomarkers such as DEF should be thoroughly verified, validated, and evaluated (following ISO9000:2008) before they can be used to enrich populations in clinical trials and aid community physicians to diagnose prodromal AD clinically. *Verification *consists in assessing that the system is built according to its specifications (i.e., assessing that the system is built correctly) and that test data is accurate. *Validation *consists in assessing that the system actually fulfills the purpose for which it was intended (i.e., assessing that the correct system was built). *Evaluation *consists in assessing that the system is accepted by the end-users and performs well for a specific purpose (i.e., assessing that the system is valuable). These are important, necessary, and often overlooked steps between bench and bedside in the research and clinical contexts.

In this study, we proposed a reliability analysis of our high-dimensional morphological metric in a large-scale multicenter setting. Reliability is a *necessary, but not sufficient, component of validity*. Our scan/repeat and scan/rescan results demonstrate that DEF is a reliable metric for medial temporal lobe atrophy estimations. 

We further estimated minimum precision threshold that must be added to the effect size to obtain true cohort sizes in the case of clinical trials. While this resulted in increased number of subjects, this increase is somewhat negligible, especially when comparing trial sizes using DEF to those obtained with other metrics, for example, ADAS-Cog [22] or MMSE [12], as mentioned in Schuff et al. [23].

While large datasets represent one of the strengths of the current study, it is not without its limitations. First is the lack of systematic pathological evaluation in both the *Classification group* and the ADNI data. The former implies that the classification function is not optimal for the task of discriminating CTRL from AD; the latter relates to the stability of the DEF. Further, while the mean and confidence intervals are relatively tight, standard deviations tend to be elevated. While it makes the DEF metric suitable for group studies, more work would be required for individual predictions. However, by design, we refrained from using techniques (e.g., within-subject registration, within-subject intensity normalization) that are specifically aimed at removing random and/or systematic errors in individual subject scanning that are not relevant to the pathology. For example, it is expected that within-subject registration would increase spatial concordance, and hence positional variability in the projected intensity and deformation spaces. Such techniques should be considered when continuing our investigations regarding the longitudinal reliability and overall validity of the DEF.

## Abbreviations

AD: Alzheimer's disease
ADNI: Alzheimer's disease neuroimaging initiative
CTRL: Control subjects
DEF: disease evaluation factor
MCI: mild cognitive impairment
MRI: magnetic resonance imaging
MTA: medial temporal lobe atrophy.

## Endnotes

1. Data used in the preparation of this paper for the *Scan/Repeat Test Group* were obtained from the Alzheimer's Disease Neuroimaging Initiative database (adni.loni.ucla.edu). The ADNI was launched in 2003 by the National Institute on Aging, the National Institute of Biomedical Imaging and Bioengineering, the Food and Drug Administration, private pharmaceutical companies, and nonprofit organizations, as a $60 million, 5-year public private partnership. The primary goal of ADNI has been to test whether serial MRI, positron emission tomography, other biological markers, and clinical and neuropsychological assessment can be combined to measure the progression of MCI and early AD. Determination of sensitive and specific markers of very early AD progression is intended to aid researchers and clinicians to develop new treatments and monitor their effectiveness, as well as lessen the time and cost of clinical trials. The Principal Investigator of this initiative is Michael W. Weiner, MD, VA Medical Center and University of California San Francisco CA, USA. ADNI is the result of efforts of many coinvestigators from a broad range of academic institutions and private corporations, and subjects have been recruited from over 50 sites across the USA and Canada. The initial goal of ADNI was to recruit 800 adults, ages 55 to 90, to participate in the research, approximately 200 cognitively normal older individuals to be followed for 3 years, 400 people with MCI to be followed for 3 years, and 200 people with early AD to be followed for 2 years. For up-to-date information, see. http://www.adni-info.org/.
